# Chronic obstructive pulmonary disease phenotypes in Turkey: the COPET study—a national, multicenter cross-sectional observational study

**DOI:** 10.55730/1300-0144.5416

**Published:** 2022-01-01

**Authors:** Esra ERTAN YAZAR, Burcu ARPINAR YİĞİTBAŞ, Can ÖZTÜRK, Mukadder ÇALIKOĞLU, Gazi GÜLBAŞ, Muzaffer Onur TURAN, Hülya ŞAHİN, Nurhan SARIOĞLU, Nevin TACİ HOCA, Fulsen BOZKUŞ, Seda TURAL ÖNÜR, Nihal Arzu MİRİCİ, Nalan OGAN, Nilgün Yılmaz DEMİRCİ, Burcu YORMAZ, Ayperi ÖZTÜRK, Filiz KOŞAR, Evrim Eylem AKPINAR, Gülşah GÜNLÜOĞLU, Burak METE, Mecit SÜERDEM

**Affiliations:** 1 Department of Chest Diseases, Faculty of Medicine, İstanbul Medeniyet University, İstanbul, Turkey; 2 Department of Chest Diseases, Faculty of Medicine, Gazi University, Ankara, Turkey; 3 Department of Chest Diseases, Faculty of Medicine, Mersin University, Mersin, Turkey; 4 Department of Chest Diseases, Faculty of Medicine, İnönü University, Malatya, Turkey; 5 Department of Chest Diseases, Faculty of Medicine, İzmir Katip Çelebi University, İzmir, Turkey; 6 Department of Chest Diseases, Dr Suat Seren Chest Disease and Chest Surgery Research and Training Hospital, İzmir, Turkey; 7 Department of Chest Diseases, Faculty of Medicine, Balıkesir University, Balıkesir, Turkey; 8 Department of Chest Diseases, Atatürk Chest Disease and Chest Surgery Research and Training Hospital, Ankara, Turkey; 9 Department of Chest Diseases, Faculty of Medicine, Kahramanmaraş Sütçü İmam University, Kahramanmaraş, Turkey; 10 Department of Chest Diseases, Yedikule Chest Disease and Chest Surgery Research and Training Hospital, İstanbul, Turkey; 11 Department of Chest Diseases, Faculty of Medicine, Çanakkale On Sekiz Mart University, Çanakkale, Turkey; 12 Department of Chest Diseases, Faculty of Medicine, Ufuk University, Ankara, Turkey; 13 Department of Chest Diseases, Faculty of Medicine, Selçuk University, Konya, Turkey; 14 Department of Public Health, Faculty of Medicine, Çukurova University, Adana, Turkey; 15 Department of Chest Diseases, Medical Park Hospital, İstanbul, Turkey

**Keywords:** ACO, chronic bronchitis, COPD, emphysema, phenotype

## Abstract

**Background/aim:**

While mortality rates decrease in many chronic diseases, it continues to increase in COPD. This situation has led to the need to develop new approaches such as phenotypes in the management of COPD. We aimed to investigate the distribution, characteristics and treatment preference of COPD phenotypes in Turkey.

**Materials and methods:**

The study was designed as a national, multicenter, observational and cross-sectional. A total of 1141 stable COPD patients were included in the analysis.

**Results:**

The phenotype distribution was as follows: 55.7% nonexacerbators (NON-AE), 25.6% frequent exacerbators without chronic bronchitis (AE NON-CB), 13.9% frequent exacerbators with chronic bronchitis (AE-CB), and 4.8% with asthma and COPD overlap (ACO). The FEV_1_ values were significantly higher in the ACO and NON-AE than in the AE-CB and AE NON-CB (p < 0.001). The symptom scores, ADO (age, dyspnoea and FEV_1_) index and the rates of exacerbations were significantly higher in the AE-CB and AE NON-CB phenotypes than in the ACO and NON-AE phenotypes (p < 0.001). Treatment preference in patients with COPD was statistically different among the phenotypes (p < 0.001). Subgroup analysis was performed in terms of emphysema, chronic bronchitis and ACO phenotypes of 1107 patients who had thoracic computed tomography. A total of 202 patients had more than one phenotypic trait, and 149 patients showed no features of a specific phenotype.

**Conclusion:**

Most of the phenotype models have tried to classify the patient into a certain phenotype so far. However, we observed that some of the patients with COPD had two or more phenotypes together. Therefore, rather than determining which phenotype the patients are classified in, searching for the phenotypic traits of each patient may enable more effective and individualized treatment.

## 1. Introduction

Worldwide, 3 million deaths were caused by chronic obstructive pulmonary disease (COPD) in 2016, and COPD causes more deaths each year than lung cancer and human immunodeficiency virus together^1^. The estimated overall population prevalence of COPD with Global Initiative for Chronic Obstructive Lung Disease (GOLD) Stage II and higher is about 10.1% [1]. Demographic variables alter the prevalence of COPD by country. The city of Adana, which is located in the southern region of Turkey, was included in the Burden of Obstructive Lung Disease (BOLD) Study and the prevalence of COPD in this city was reported at an alarmingly high level of 19% [1]. However, another epidemiologic study investigating cases of physician-diagnosed COPD in the Turkish population in 2016 revealed the prevalence of COPD as 5.8%. Additionally, the overall prevalence rates increased from 4.3% in 2012 to 5.8% in 2016, which was a 35% relative rise [2].

In the last two decades, research on the determination of phenotypes in COPD to improve treatment efficacy has increased enormously. However, some difficulties are still encountered while determining phenotypes: 1. COPD can be defined as a complex and heterogeneous disease with several components, as there is a nonlinear dynamic interaction between them (complex), not all these components may be present in all patients, and they may not be seen at all times in one patient (heterogeneous) [3]. 2. The disease characteristics that can be used to define phenotypes in COPD include clinical features, imaging, pulmonary function tests and biomarkers, but integrating these various characteristics with the aim of determining phenotypes is challenging. 3. Even the most consensual phenotypes such as chronic bronchitis, asthma and COPD overlap (ACO), or emphysema may not be easy to manage, since considerable overlaps can be seen between them.

We aimed primarily to investigate the distribution of COPD phenotypes in our study population from Turkey and to compare the demographics, clinical characteristics and pharmacological treatment of the patients according to their phenotypes. The secondary aim of the study was to discuss the challenges we encountered in dividing patients into phenotypes. To the best of our knowledge, this is the first multicenter study presenting the distribution of COPD phenotypes in Turkey.

## 2. Material and methods

### 2.1. Study design and patients

**Figure 1:**
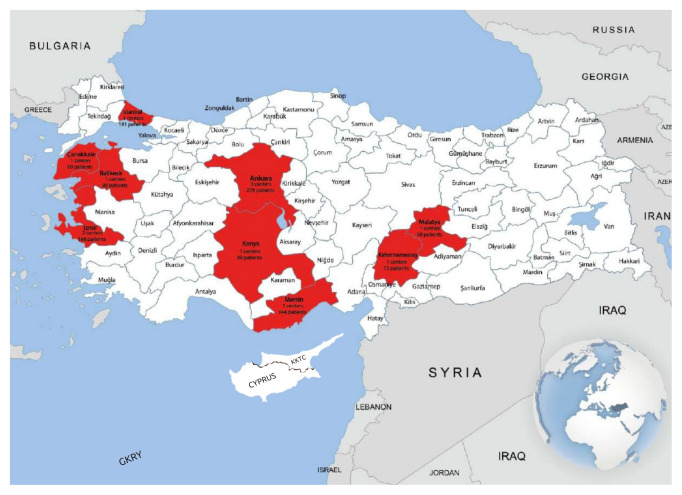
The centers and the number of patients participating in the study.

Chronic obstructive pulmonary disease phenotypes in Turkey “The COPET Study” was designed as a national, multicenter, observational and cross-sectional study. The patients were enrolled prospectively between December 2018 and January 2020. Twelve centres (Yedikule Chest Disease and Chest Surgery Research and Training Hospital, İstanbul; Gazi University, Ankara; Mersin University, Mersin; İnönü University, Malatya; İzmir Katip Çelebi University, İzmir; Dr Suat Seren Chest Disease and Chest Surgery Research and Training Hospital, İzmir; Balıkesir University, Balıkesir; Atatürk Chest Disease and Chest Surgery Research and Training Hospital, Ankara; Kahramanmaraş Sütçü İmam University, Kahramanmaraş; Çanakkale On Sekiz Mart University, Çanakkale; Ufuk University, Faculty of Medicine, Ankara; Selçuk University, Konya) which may reflect our country in general and 20 researchers who are pulmonary specialists participated in the study. The center and the number of patients participating in the study are shown on the map of Turkey in Figure 1. A sample size of 1145 achieves 99% power to detect an effect size (W) of 0.2052 using a 9 degrees of freedom chi-square test with a significance level (alpha) of 0.001 [4]. The study was approved by the ethics committee of Ufuk University School of Medicine (no. 20190328/4), and written informed consent was obtained from each patient.

Patients who met the inclusion criteria were consecutively recruited in the study when they visited hospital-based pulmonary outpatient clinics. The inclusion criteria were as follows: ≥ 40 years of age, COPD diagnosis for at least a year and confirmed diagnosis of COPD with a postbronchodilator forced expired volume in 1 s (FEV_1_)/forced vital capacity (FVC) < 0.7, current/exsmoker (≥ 10 pack-year smoking history) or a nonsmoker with at least 10 years’ biomass exposure. Patients who did not have a definitive diagnosis of COPD, had COPD exacerbation within the past 6 weeks prior to enrolment, were unable to complete the case report form or who had chronic respiratory diseases (other than noncystic fibrosis bronchiectasis, tuberculosis sequelae and asthma) were excluded from the study. The patients’ demographic and clinical characteristics were obtained by face-to-face interviews with the patients and from hospital records.

Pulmonary function tests (PFT) and haemograms were performed if these were not in the patients’ hospital records within the last 6 months. The PFT were obtained using standard equipment according to the American Thoracic Society/European Respiratory Society consensus guidelines [5]. The presence of emphysema in thorax computed tomography (CT) was used to quantify the extent of emphysema at −950 Hounsfield units and was confirmed using the local radiologists [6]. COPD exacerbation was defined as patient reports of increased symptoms requiring treatment with systemic steroids and/or antibiotics with or without admission to the emergency department and/or hospitalization^2^. We recorded only moderate and severe exacerbations.

We used the POPE study’s algorithm to determine the COPD phenotypes which is rationale and methodology of a study to phenotype patients with COPD in Central and Eastern Europe in a real-life setting [7]. 1) patients who met the ACO criteria were considered to have the ACO phenotype; 2) patients with few than two exacerbations (not requiring hospitalization) in the previous year were classified as the non-exacerbator phenotype (NON-AE); 3) exacerbators with self-reported chronic cough and expectoration for more than three months of the year over two consecutive years were described as frequent exacerbators with chronic bronchitis (AE-CB); and 4) the remaining exacerbators were classified as frequent exacerbators without chronic bronchitis (AE NON-CB) [7]. ACO was diagnosed when two major criteria or one major and two minor criteria were met different from POPE Study. The major criteria included a personal history of asthma, a positive bronchodilator test (increase in FEV_1_ ≥ 15% and ≥ 400 mL) and eosinophilia in the sputum; the minor criteria included a personal history of atopy, high total IgE, and a positive bronchodilator test (increase in FEV_1_ ≥ 12% and ≥ 200 mL) [8]. We used peripheral blood eosinophilia (> 300 cells per mm^3^) a surrogate marker for sputum eosinophilia.

### 2.2. Measurements

Dyspnoea was assessed based on the modified Medical Research Council (mMRC) dyspnoea scale [9] and also symptom status was evaluated using the COPD Assessment Test (CAT) [10]. The postbronchodilator PFT values of each patient were recorded, and COPD severity was classified by the predictive FEV_1_ values: stage 1 (mild), FEV_1_ ≥ 80%; stage 2 (moderate), FEV_1_ ≥ 50% and < 80%; stage 3 (severe), FEV_1_ ≥ 30% and < 50%; and stage 4 (very severe), FEV_1_ < 30% according to the GOLD 2021 recommendations^3^. The presence of comorbidities was evaluated by the Charlson comorbidity index (CCI) [11]. For each patient, we calculated the age, dyspnoea and airflow obstruction (ADO) index, which combines age, the mMRC score and the FEV_1_. The total score of the ADO index ranges from zero to 10 points, and a higher score indicates worse prognosis in patients with COPD [12]. All the patients were divided into four risk/symptom categories, according to the GOLD 2021 recommendations: low risk and fewer symptoms (category A); low risk and more symptoms (category B); high risk and fewer symptoms (category C); and high risk and more symptoms (category D). Based on this categorization, the cut-off points for risk were exacerbations in the previous year ≥ 2 or ≥ 1 leading to hospitalization, and the cut-off points for more symptoms were CAT scores ≥ 10 and/or mMRC ≥ 2^3^.

### 2.3. Statistical analysis

SPSS 22.0.0 package program (IBM Corporation, Armonk, NY, USA, 2013) was used in the analysis of the data. Categorical data were given as number and percentage; quantitative data were given as median, mean and standard deviation.

Kolmogorov–Smirnov test was used as normal distribution test. Kruskal–Wallis test and Mann–Whitney U test were used in the analysis of quantitative data, and Pearson chi-square analysis test was used in the analysis of categorical data. In case the minimum expected value was below 1 in chi-square analyses or the expected cell number below 5 was more than 20%, exact correction had been made. Column comparison was used for posthoc analysis in categorical data, and Dunn test was used for quantitative data. A value of p < 0.05 was considered significant.

## 3. Results

### 3.1. Demographic and clinical features

A total of 1203 patients were recruited from the study centers, and 62 patients were excluded because they had missing data or did not meet the inclusion criteria. A total of 1141 stable COPD patients with a mean age of 65.8 ± 9 years were included, of whom 87.2% (n = 995) were male. The rates and numbers of current smokers, exsmokers and nonsmokers were 32.2% (n = 367), 62.9% (n = 718), and 4.9% (n = 56), respectively. The mean biomass exposure of all the patients was 34.9 ± 17.6 years. According to the CCI, 41.1% of patients had at least one comorbidity, and the three most common comorbidities were heart failure, diabetes mellitus (DM) and myocardial infarction. The demographic and clinical features of the patients are given in Table 1.

### 3.2. Distribution of phenotypes, GOLD categories and stages

**Figure 2:**
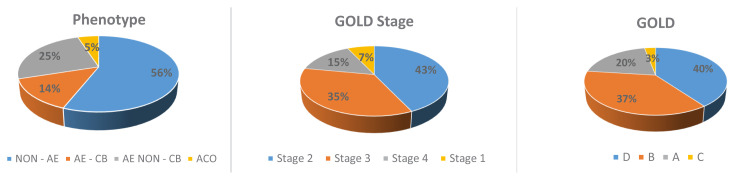
Distribution of phenotypes, GOLD stages and GOLD categories (A–D) in COPD patients. Abbreviations: NON-AE, nonexacerbators; AE NON-CB, frequent exacerbators without chronic bronchitis; AE-CB, frequent exacerbators with chronic bronchitis; COPD, chronic obstructive pulmonary disease; ACO, asthma-COPD overlap; GOLD, Global initiative for chronic obstructive lung disease; FEV_1_, forced expiratory volume in 1 s (% predicted).

The NON-AE phenotype was detected at the highest rate (55.7%; n = 635), followed by the AE NON-CB (25.6%; n = 292), the AE-CB (13.9%; n = 159) and the ACO phenotypes (4.8%; n = 55). The highest rate of GOLD stage was 2 (42.6%), and the highest GOLD category was D (39.7%). The distribution of the phenotypes, the GOLD categories (A–D) and the GOLD stages are shown in Figure 2.

### 3.3. Demographic, clinical and laboratory characteristics of the patients according to the phenotypes

There were statistically significant differences between the phenotypes in terms of the PFT values, the CAT and the mMRC scores, the ADO index, history of smoking, exacerbation and the eosinophil count and percentage. The FEV_1_ value was significantly higher in the ACO and NON-AE phenotypes than in the AE-CB and AE NON-CB phenotypes (p < 0.001). The eosinophil count and percentages were the highest in the ACO phenotype (p < 0.001). The CAT and the mMRC scores, the ADO index and the rates of exacerbations in the previous year were significantly higher in the AE-CB and NON-CB phenotypes than in the ACO and NON-AE phenotypes (p < 0.001). Emphysema was highest in the AE NON-CB phenotype. There was no difference between the phenotypes in terms of age, body mass index (BMI), biomass exposure and the mean number of comorbidities. However, only DM was highest in the AE-CB phenotype (p < 0.001). The characteristics of the COPD patients according to phenotype are given in Table 2.

### 3.4. Pharmacological treatment usage rates by COPD phenotypes

Long-acting muscarinic antagonists (LAMAs) were preferred more in the NON-AE phenotype, long-acting β-agonists (LABAs) in the ACO and NON-AE phenotypes, LABA + inhaled corticosteroid (ICS) in the ACO phenotype and LABA + LAMA + ICS (triple therapy) in the AE-CB and AE NON-CB phenotypes (Table 3).

### 3.5. Venn diagrams of COPD

**Figure 3:**
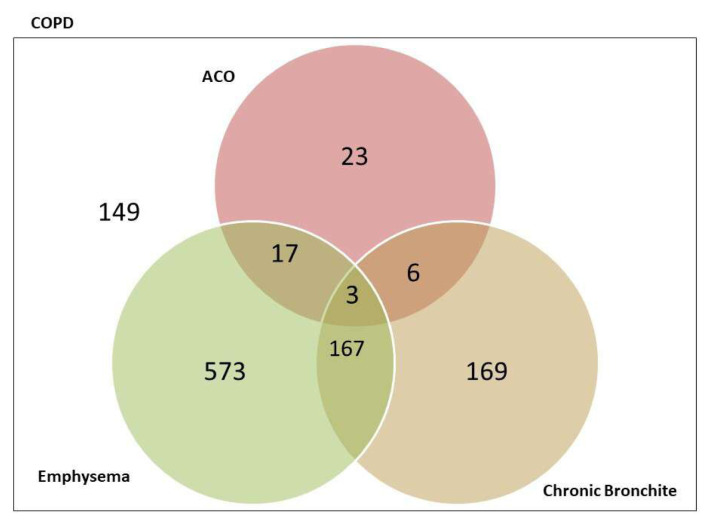
Venn diagram of chronic obstructive pulmonary disease (COPD). The total number of patients 1107. ACO, asthma-COPD overlap.

The patients with thorax CT (n = 1107) are shown in a Venn diagram. Seven hundred and sixty patients (68.7%) had emphysema, 345 patients (31.2%) had chronic bronchitis, and 49 patients (4.2%) had ACO, according to the clinical, laboratory and radiological characteristics. A total of 149 patients (13.5%) did not have any of these phenotypes (Figure 3).

## 4. Discussion

This study indicates that the NON-AE phenotype is the most prevalent (55.7%), followed by the AE NON-CB (25.6%), AE-CB (13.9%) and ACO (4.8%) phenotypes. NON-AE is also the most commonly observed COPD phenotype in other countries of the world [4,13–15]. While AE NON-CB was the second most common phenotype in our study, it was reported to be the AE-CB phenotype in other regions [4,13–15]. This difference may be caused by genetic or environmental factors. The ACO phenotype was observed at the lowest rate (4.8%), which is in concordance with other countries, as ACO rates ranged from 5% to 15% in separate studies [4,13–15]. We used the POPE study’s criteria, which suggests categorizing patients with frequent exacerbations into two phenotypes: AE-CB and AE NON-CB [6]. We found the highest rate of emphysema among the AE NON-CB (86%) phenotype. This indicated that the predominant condition in the AE NON-CB phenotype was emphysema. In our study, almost 3.5% of the patients who were frequent exacerbators did not have chronic bronchitis or emphysema. Similarly, a small proportion of patients (2.3%) remained unclassified in the CHAIN Cohort [16]. Hence, although this phenotype algorithm is simple, clinically relevant and easily applicable, it may not fully meet the needs of some patients.

There were statistically significant differences amongst the phenotypes in terms of clinical laboratory and radiological characteristics in our study. The patients with the AE-CB and NON-CB phenotypes were more symptomatic and had worse lung function parameters, similar to the POPE study’s results [17]. The ADO index, which seems to have medium- and long-term predictive prognostic reliability [12], was higher in the AE-CB and the NON-CB phenotypes than in the ACO and NON-AE. The rates of exacerbations in the previous year were significantly higher in the AE-CB and NON-CB phenotypes compared to the other phenotypes. Our results are similar to the literature; in the PLATINO Study, the subjects with chronic bronchitis had worse lung function and general health status and more respiratory symptoms, physical activity limitation and exacerbations [18]. Frequent exacerbators with the chronic bronchitis phenotype were the most symptomatic patient, with a higher BODE (BMI, airway obstruction, dyspnea, exercise capacity) score, in the CHAIN Cohort [16]. The number of current smokers in the NON-AE phenotype, the exsmokers in the frequent exacerbators phenotype and the nonsmokers in the ACO phenotype were significantly higher compared to the other phenotypes. The quantity of cigarettes smoked (pack-years) was significantly higher in the AE NON-CB than in the ACO and NON-AE in our study. Similarly, the Polish subcohort of the POPE study found that smoking habits differ among the phenotypes. There were fewer current smokers in the AE-CB compared to the ACO and NON-AE phenotypes, and the AE-CB phenotype group smoked more cigarettes compared to the ACO phenotype group [13]. The POPE study showed that patients with ACO were younger on average, had a higher BMI and were more likely to be female compared to the other phenotypes [17]; we found no difference between the phenotypes in terms of age, sex, BMI and biomass exposure. Previous study found that 97.7% of COPD patients had one or more reported comorbidities [19]. We detected one comorbidity in at least 41.1% of the subjects, according to the patients’ self-reports. This difference may be a result of unrecorded some common comorbidities in our study, such as hypertension, anxiety and depression, which are not included in the CCI. There were no differences amongst the phenotypes in terms of the mean number of comorbidities, but DM was the highest in the AE-CB phenotype in our study. Similarly, comorbidities were measured by the CCI in both the CHAIN cohorts and the Polish subcohort of the POPE study, and there were no differences amongst the phenotypes [13,16].

We found that the pharmacological treatment preferences for COPD were statistically different between the phenotypes. The most commonly observed agents were bronchodilators in the NON-AE, LABA + ICS in the ACO and triple inhaler therapies in the frequent exacerbator phenotypes. Even though the patients in our cohort were not treated according to their phenotypes, the physicians seemed to take into account phenotype-based therapy in clinical practice. By contrast, 29.1% of patients with ACO were not receiving ICS, despite the benefits that ICS has demonstrated in this group of patients. Similarly, in other countries, it has been observed that treatment patterns differ among the phenotypes and ICS has not been used as widely as expected in the ACO phenotype [14,16,17].

We performed subgroup analysis in 1107 patients with thorax CT, 202 patients (18.3%) had two or more phenotypic features (emphysema ± chronic bronchitis ± ACO). Our results support the idea that the phenotypes could coexist in some patients at the same time. In contrast, 149 patients (13.5%) could not be classified by any phenotype. We think that some phenotypes caused by pathophysiological changes such as emphysema, chronic bronchitis, or bronchiectasis are permanent (they may be categorized as fixed phenotypes), while some phenotypes, such as frequent exacerbator or more symptomatic phenotypes, which may change over the course of the disease (they may be categorized as variable phenotypes). In follow-up, evaluating fixed and variable phenotypes together in each patient may enable more effective management, not only for those with frequent exacerbations but also for patients with phenotypes that are more symptomatic without frequent exacerbation.

This study has some limitations. First, all the centers participating in the study were pulmonary outpatient clinics of a university or training hospital, which may have led to the recruitment of more severe patients. However, as there is no referral chain to tertiary care hospitals in our country, patients with stage 1 and category A could also have been enrolled in the study. Also, our results may not reflect Turkey in general, however, we think that the inclusion of big cities such as İstanbul, Ankara and İzmir, which have received immigration from all over the country, reduces this bias. Second, the patients’ PFTs and laboratory and radiology results were recorded retrospectively, if present, in their previous follow-ups. Finally, the study had a cross-sectional design, meaning that the patients’ medical histories, such as exacerbations, comorbidities and exposures were recorded retrospectively based on patient statements and hospital records, which may potentially lead to recall bias.

In conclusion, this study provides important data for future studies regarding the distribution, characteristics and pharmacological treatment of predefined COPD phenotypes in the Turkish population. Additionally, our results support the fact that there are some dilemmas when defining COPD phenotypes. We consider that, rather than determining which phenotype the patients are classified in, searching for the phenotypic characteristics of each patient may enable more effective and individualized use of pharmacological and nonpharmacological treatment options.

## Figures and Tables

**Table 1:** Demographic and clinical characteristics of the patients (n = 1141).

Variables	n (%) or mean ± SD
**Age (years)**	65.8 ± 9.0
**Sex, male**	995 (87.2)
**BMI (kg/m** **^2^** **)**	25.5 ± 4.8
**Smoking (package-year)**	46.7 ± 24.0
**Biomass exposure**	496 (43.5)
**The time since COPD diagnosis (years)**	6.2 ± 4.7
**FEV** **_1_** **% predicted**	50.9 ± 19.4
**FVC % predicted**	67.6 ± 20.2
**FEV** **_1_** **/FVC**	56.3 ± 9.8
**CAT score**	16.4 ± 9.0
**mMRC scor**e	2.0 ± 1.0
**More symptomatic according to mMRC or CAT**	875 (76.7)
**Number of exacerbations in the previous year**	1.4±2.2
**Number of comorbidities**	0.6±0.8
**The patients with at least one comorbidity**	469 (41.1)
**The patients with CHF**	167 (14.6)
**The patients with DM**	152 (13.3)
**The patients with MI**	105 (9.2)
**The patients with malignancy**	69 (6.0)
**Eosinophil count 10** **^3^** **/mL**	199.9 ± 191.6
**ADO index**	4.1 ± 1.7

Abbreviations: BMI, body mass index; FEV_1_, forced expiratory volume in 1 s; FVC, forced vital capacity; COPD, chronic obstructive pulmonary disease; CAT, COPD assessment test; mMRC, modified Medical Research Council; GOLD, Global initiative for chronic obstructive lung disease; CHF, congestive heart failure; DM, diabetes mellitus; MI, myocardial infarction; ADO index, age, dyspnea, airflow obstruction index.

**Table 2:** Comparison of the COPD phenotypes in terms of demographic, clinical and laboratory characteristics.

	COPD phenotypes				
	ACO n = 55; 4.8%	NON-AE n = 635; 55.7%	AE-CB n = 159; 13.9%	AE NON-CB n = 292; 25.6%	p value
**Variables**	Med (IQR)	Med (IQR)	Med (IQR)	Med (IQR)	
**Age (years)**	65 (9.3)	66 (12)	65 (11.5)	66 (14)	0.138
**Education (years)**	5 (6)	5 (3)	5 (3)	5 (2.5)	**0.012**
**Smoking (package-year)**	46.5 (25.8)	45 (25.5)	40 (25)	48 (27.5)	**0.001**
**BMI (kg/m** **^2^** **)**	23.5(6.7)	25.5(6)	25 (4.6)	26 (6.8)	0.331
**Biomass exposure (year)**	38.5(39.5)	36(32)	40 (40)	30 (30)	0.405
**The time since COPD diagnosis (years)**	5 (4.8)	4 (4.5)	5 (6)	5 (7)	**<0.001**
**FEV** **_1_** **% predicted**	54 (26.2)	57 (27.1)	40 (27)	40 (22)	**<0.001**
**CAT score**	14 (12)	12(12)	21 (13)	18 (16)	**<0.001**
**mMRC score**	2 (1)	2 (1)	3 (1)	2 (1)	**<0.001**
**Eosinophil count 10** **^3^** **/mL**	200 (520)	176 (182.5)	160 (240)	130 (180)	**<0.001**
**Eosinophil %**	2,7 (3.9)	2 (2.2)	1.8 (2.4)	1.6 (2.4)	**<0.001**
**Number of comorbidities**	0 (1)	0 (1)	0 (1)	0 (1)	0.300
**Number of exacerbations in the previous year**	2 (3.5)	0 (1)	2 (1)	2 (2)	**<0.001**
**ADO index**	4 (3.0)	4 (3.0)	5 (2.0)	5 (2.0)	**<0.001**
**n, (%)**					
**Smoker**	15 (27.3)^a,b^	243 (38.3)^b^	36 (22.6)^a^	73 (25.0)^a^	**<0.001**
**Exsmoker**	35 (63.6)^a,b^	370 (58.3)^b^	112 (70.4)^a^	201 (68.8)^a^	**<0.001**
**Nonsmoker**	5 (9.1)^a^	22 (3.5)^b^	11 (6.9)^a,b^	18 (6.2)^a,b^	**<0.001**
**Emphysema**	20 (40.8)^a^	403 (65.1)^b^	91 (59.5)^b^	246 (86.0)^c^	**<0.001**
**Bronchiectasis**	11 (22.4)^a^	151 (24.4)^a^	42 (27.5)^a^	75 (26.2)^a^	0.806
**Hospitalization in the previous year**	17 (30.9)^a^	0 (0)^b^	111 (69.8)^c^	193 (66.1)^c^	**<0.001**
**ICU admission in the previous year**	8 (14.5)^a^	0 (0)^b^	39 (24.5)^a^	59 (20.2)^a^	**<0.001**
**ED admission in the previous year**	26 (47.3)^a^	107 (16.9)^b^	130 (81.8)^c^	232 (79.5)^c^	**<0.001**
**DM**	3 (5.5) ^a^	71 (11.2) ^a^	37 (23.3) ^b^	41 (14.0) ^a^	**<0.001**

Abbreviations: ACO, asthma-COPD overlap; NON-AE; nonexacerbators; AE-CB, frequent exacerbators with chronic bronchitis; AE NON-CB, frequent exacerbators without chronic bronchitis; BMI, body mass index; FEV_1_, forced expiratory volume in 1 s; FVC, forced vital capacity; COPD, chronic obstructive pulmonary disease; CAT, COPD assessment test; mMRC, modified Medical Research CounciI; GOLD, Global initiative for chronic obstructive lung disease; ICU, intensive care unit, ED, emergency department; DM, diabetes mellitus. Statistically significant differences between phenotypes were shown as a superscript with symbols a, b, c, d.

**Table 3:** Pharmacological treatment usage rates by COPD phenotypes.

	*COPD Phenotypes*				
*Inhalers drugs* n (%)	ACO n = 55 (4.8%)	NON-AE n = 635 (55.7%)	AE-CB n = 159 (13.9%)	AE NON-CB n = 292 (25.6%)	p
**LAMA**	5 (9.1)^a,b^	110 (17.3)^b^	7 (4.4)^a^	14 (4.8)^a^	
**LABA**	5 (9.1)^a^	67 (10.6)^a^	3 (1.9)^b^	6 (2.1)^b^	
**LAMA+LABA**	6 (10.9)^a,b,c^	109 (17.2)^c^	7 (4.4)^b^	31 (10.6)^a^	
**LABA+ICS**	23 (41.8)^a^	102 (16.1)^b^	34 (21.4)^b^	52 (17.8)^b^	**<0.001**
**LAMA+LABA+ICS**	15 (27.3)^a^	230 (36.2)^a^	102 (64.2)^b^	179 (61.3)^b^	
**SABA+SAMA+ICS**	1 (1.8)^a^	11 (1.7)^a^	6 (3.8)^a^	10 (3.4)^a^	
**SABA+SAMA**	0 (0.0)^a^	6 (0.9)^a^	0 (0.0)^a^	0 (0.0)^a^	
***Additional drugs***					
**Theophyllin**	3 (42.9)^a^	31 (50.0)^a^	12 (60.0)^a^	37 (53.6)^a^	
**Mucolytic**	0 (0.0)^a^	25 (40.3)^b^	1 (5.0)^a^	26 (37.7)^b^	
**Roflumilast**	0 (0.0)^a^	1 (1.6)^a^	1 (5.0)^a^	1 (1.4)^a^	**<0.001**
**Mucolytic + Theophyllin**	1 (14.3)^a,b,c^	4 (6.5)^c^	6 (30.0)^b^	5 (7.2)^a,c^	
**LTA**	3 (42.9)^a^	1 (1.6)^b^	0 (0.0)^b^	0 (0.0)^b^	

Abbreviations: COPD, chronic obstructive pulmonary disease; ACO, asthma-COPD overlap; NON-AE, nonexacerbators; AE-CB, frequent exacerbators with chronic bronchitis; AE NON-CB, Frequent exacerbators without chronic bronchitis; LAMA, long acting muscarinic antagonist; LABA, long acting beta agonist; ICS: inhaled corticosteroid; SABA, short acting beta agonist; SAMA, short acting muscarinic antagonist; LTA, leukotriene antagonist. Data are presented as number (%). Statistically significant differences between phenotypes were shown as a superscript with symbols a, b, c, d.

## Footnotes

1 World Health Organization (2016). Global health estimates [online]. Website http://www.who.int/news-room/fact-sheets/detail/chronic-obstructive-pulmonary-disease-(copd) (Accessed November 2018).

2 GOLD (2018). Global Strategy for the Diagnosis, Management and Prevention of COPD, Global Initiative for Chronic Obstructive Lung Disease (GOLD) [online]. Website https://goldcoped.org/ (Accessed July 17, 2018).

3 Global Initiative for Chronic Obstructive Lung Disease (GOLD) (2021). Global strategy for the diagnosis, management, and prevention of chronic obstructive pulmonary disease 2021 report [online]. Website https://goldcopd.org/wp-content/uploads/2020/11/GOLD-REPORT-2021-v1.1-25Nov20_WMV.pdfgoldcopd.org/gold-reports/ (Available November 2021)
